# Estimation in emerging epidemics: biases and remedies

**DOI:** 10.1098/rsif.2018.0670

**Published:** 2019-01-16

**Authors:** Tom Britton, Gianpaolo Scalia Tomba

**Affiliations:** 1 Department of Mathematics, Stockholm University, 10691 Stockholm, Sweden; 2 Department of Mathematics, University of Rome Tor Vergata, 00133 Rome, Italy

**Keywords:** statistics, emerging epidemic, estimation bias, basic reproduction number

## Abstract

When analysing new emerging infectious disease outbreaks, one typically has observational data over a limited period of time and several parameters to estimate, such as growth rate, the basic reproduction number *R*_0_, the case fatality rate and distributions of serial intervals, generation times, latency and incubation times and times between onset of symptoms, notification, death and recovery/discharge. These parameters form the basis for predicting a future outbreak, planning preventive measures and monitoring the progress of the disease outbreak. We study inference problems during the emerging phase of an outbreak, and point out potential sources of bias, with emphasis on: contact tracing backwards in time, replacing generation times by serial intervals, multiple potential infectors and censoring effects amplified by exponential growth. These biases directly affect the estimation of, for example, the generation time distribution and the case fatality rate, but can then propagate to other estimates such as *R*_0_ and growth rate. We propose methods to remove or at least reduce bias using statistical modelling. We illustrate the theory by numerical examples and simulations.

## 1. Introduction

During the last decades, several new disease outbreaks have caused worldwide alarm, e.g. SARS, foot and mouth disease, H1N1 influenza, and, more recently, Ebola. What these outbreaks have in common is the need for estimation of key parameters early on, in order to plan interventions and monitor the progress of the disease. Thus estimation must be performed in the *emerging* phase of an outbreak, when the *number* of infected individuals is in the hundreds or at most thousands, while the community *fraction* of infected is still small. Typically, the early numbers grow exponentially, as also predicted by mathematical epidemic models [1].

There may be many complicating or limiting factors related to incompleteness of data, lack of detailed knowledge about the disease and other issues when analysing data from the early phase of an outbreak. Despite these complicating factors, the conclusions drawn from early analyses, often based on simple models, are usually highly valuable. The aim of the present paper is to identify and highlight some of the potential biases in the statistical analysis of emerging outbreaks and to illustrate how they can propagate to parameter estimates and predictions. A further aim is to give some fairly simple suggestions for how to reduce, or even remove, such biasing effects.

The typical available data consist of reported numbers of cases per day or week, some case histories illustrating the course of the disease and some contact tracing data containing information about possible durations between onset of symptoms of infected individuals and their infectors. The epidemic models used in the statistical analyses are often of simple form, neglecting various heterogeneities. The use of simple models in these situations is motivated by the lack of detailed information but has also recently been studied [2], showing that neglecting population structures when making inference in emerging outbreaks has little effect. However, estimation in simple models can still be quite complicated. The complications are mainly due to three factors: (1) important events, such as times of infection, are usually unobserved, but instead some proxy measures such as onset of symptoms are available, (2) estimation of parameters of the epidemic process is based on observations up to some fixed time, implying that events occurring later are censored and (3) the population of infectives is increasing (exponentially) with time.

In our investigation, we assume a simple, homogeneously mixing, model of an outbreak (the model would have to be different if one had knowledge of household size distribution, contact network, spatial heterogeneity, etc.) and we first discuss the effects of estimating the generation time distribution from observations of generation times observed backwards in time using contact tracing, i.e. the time between the infection time of an individual (the infectee) and that of his/her infector (rather than the infection time of the individuals he/she infects). The second problem we study is the effect of replacing generation times (the time between infections of an infector and an infectee) with the more commonly observed serial intervals (the time between onset of symptoms of an infector and an infectee). A third problem we discuss is how to treat the common situation, when contact tracing, of finding more than one potential infector of a case, with the implication that the backward generation time or incubation time (time from infection to symptoms) is one out of several possible values. As it turns out, the potential biasing effects of these problems all go in the same direction and, for example, the basic reproduction number *R*_0_ (the average number of infections in a fully susceptible population) can be quite significantly underestimated. We also point out some general stability properties of ratios during exponential growth that may be useful for inference. We then quantify the various biases that can arise in realistic parameter settings, both theoretically and by simulation, mostly using parameters freely adapted from the recent Ebola epidemic in West Africa [3].

In the next five sections, we describe the theory behind our results. In §7, we illustrate our findings with numerical examples and report some interesting simulation results. Section 8 is a brief discussion and, finally, mathematical details and proofs as well as detailed numerical and simulation results are collected in the electronic supplementary material. Computer code for simulations is available in separate files.

## 2. The underlying model and some key epidemiological quantities

We start by presenting the basic underlying epidemic model. We assume that individuals are at first susceptible and later may become infected, and that infected individuals may then infect other individuals. The infection ends with death or recovery. The population is assumed to be a homogeneously mixing community of homogeneous individuals. Since we model the initial phase of the outbreak, the depletion of susceptibles is considered as negligible. Also, we assume that individuals do not change their behaviour over the considered time period as a consequence of the ongoing outbreak, nor are there yet any control measures put in place by health authorities or similar. Finally, we assume that there are no seasonal changes in transmission. Predictions are made assuming that the disease spreading mechanism continues unaltered, reflecting what would presumably happen in the absence of control measures (these predictions should then be compared with predictions including various preventive measures).

Traditionally, the population effects of such an infection have been modelled using compartmental models with separate states, for example, susceptible, latent (historically called exposed and hence abbreviated by E), infectious or recovered/removed individuals (SI, SEI, SIR and SEIR models; e.g. [1,4]). Recently, modelling has reverted to something akin to the original Kermack & McKendrick [5] formulation, with emphasis on one single quantity, *β*(*s*), the average rate at which an infected individual infects new individuals *s* time units after his/her time of infection, denoted the infection rate or infectivity function. The assumption of a homogeneous community implies that *β*(*s*) is the same for all individuals, and the assumptions of no depletion of susceptibles, no preventive measures and no seasonal effects imply that *β*(*s*) is independent of the time of infection of the individual. The previously mentioned compartmental models can all be translated to this framework. While the original treatment of the Kermack–McKendrick model was deterministic (Volterra-type integral equations), statistical modelling requires a stochastic formulation which, in this case, corresponds to a Crump–Mode–Jagers branching process [6] in the initial phase of spread. It should be noted that the infectivity functions in a stochastic model may be different from individual to individual, although the average behaviour is the same, and that different stochastic models may have the same average behaviour [7].

The average infection rate *β*(*s*) completely determines the basic reproduction number *R*_0_ (the average number of new infections caused by one infectious individual in a completely susceptible population), as is well known in epidemic modelling [1] and branching process theory [7].

The basic reproduction number *R*_0_ is given by

2.1 $$R_{0}=\int_{0}^{\infty }\beta (s)\;\text{d}s.$$

It is well known that an epidemic can take off if and only if *R*_0_ > 1, which we from now on assume.

Another important quantity is the so-called *generation time* distribution *f*_G_(*s*), which is simply the infection rate scaled to make it a probability density:

2.2 $$f_{G}(s)=\frac{\beta (s)}{R_{0}}=\frac{\beta (s)}{\int_{0}^{\infty }\beta (u)\;\text{d}u}.$$

The generation time distribution is the probability distribution of the time between the moment of infection of a randomly chosen infective and that of his/her infector.

In what follows we will write *R*_0_*f*_G_(*s*) instead of *β*(*s*).

Let *i*(*t*) denote the expected incidence at *t* (time since the start of the outbreak, assumed to have been started by one infective individual), i.e. the average community rate of new infections. Since we assume that individuals infected *s* time units ago (at time *t* − *s* if present time equals *t*) will infect new individuals at rate *R*_0_*f*_G_(*s*) we have the following renewal equation for *i*(*t*) (see [1, p. 212]):

2.3 $$i(t)=\int_{0}^{t}i(t-s)R_{0}f_{G}(s)\;\text{d}\;s+R_{0}f_{G}(t)=\int_{0}^{t}R_{0}f_{G}(t-s)i(s)\;\text{d}\;s+R_{0}f_{G}(t).$$

The additive term in the above equation derives from the initial infective that is supposed to have started the outbreak at *t* = 0. It is well known that the incidence *i*(*t*) will quickly approach exponential growth *i*(*t*) ∼ *C* e^*rt*^, where *r* is the so-called Malthusian parameter, the exponential growth rate, defined as the solution to the Euler–Lotka equation

2.4 $$1=\int_{0}^{\infty }{\text{e}}^{-rt}R_{0}f_{G}(t)\;\text{d}\;t.$$

**Figure 1.**
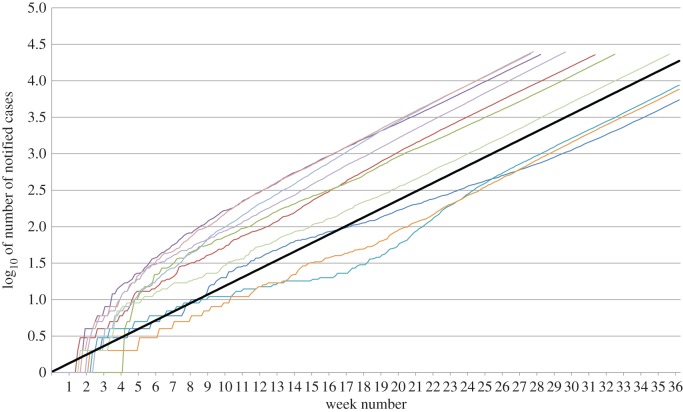
Initial stages of 10 simulated epidemics with *R*_0_ = 1.7 and generation time *G* being Gamma distributed with mean = 15 days and standard deviation = 8.7 days, resulting in *r* = 0.03873. Cumulative incidence of notified cases over time in log-scale. Black line represents expected slope (line with equation *y* = *rx*). It is seen that incidence grows exponentially (linear on log-scale) but that there is a random time-shift before the epidemic takes off in the different simulations. As explained in the electronic supplementary material, simulations were continued until 4500 cumulated cases (=3.65 in log-scale) and then for further six weeks or until week 36. (Online version in colour.)

To simplify matters, we will assume that this exponential growth of new cases holds from the start. The validity of this assumption will be shown by subsequent simulations. In figure 1, 10 simulated epidemics are plotted over time showing the exponentially growing feature (clearly visible on the log-scale).

Thus, knowing the generation time distribution $f_{G}(\cdot )$ and one of *R*_0_ and *r* allows the determination of the other one (cf. [8]), e.g. using equation (2.4). For this reason, estimation of the generation time distribution $f_{G}(\cdot )$ becomes paramount in this model formulation and will be extensively discussed in following sections. Also, various rather general conclusions about the effects of varying the components of (2.4) related to the directions of biases in the estimation of these components can be drawn. The mathematical details are given in the electronic supplementary material and the specific results will be discussed in the relevant sections.

There are other approaches to the estimation of *R*_0_ and *r*. The exponential growth rate can be directly estimated from case data and *R*_0_ through modelling approaches (e.g. [9–12]), assuming the generation time distribution to be known, or in the so-called ‘First Few Hundred’ studies, usually restricted to transmission in households (e.g. [13–15]). In [16], the joint estimation of *R*_0_ and the generation time distribution is contemplated, but the authors suggest that these methods do not work well during the early phase of an epidemic. In this paper, we assume that the generation time distribution, as well as the incubation time distribution and also distributions of time from notification to recovery/death, will be estimated by limited contact tracing or specific samples during the initial phase of the disease spread and that, otherwise, just counts of cases in various stages are available.

In the model description above, the expected incidence *i*(*t*) is a time-continuous deterministic function. The true incidence is, of course, integer-valued and, in most situations, observations are not made continuously but are aggregated in discrete time units such as days or weeks. A related discrete time model is obtained by suitably discretizing equation (2.3) so that the expected incidence *I*(*t* + 1) in time (interval) *t* + 1 is expressed as

2.5 $$I(t+1)=\sum_{s=1}^{t}I(t+1-s)R_{0}p_{G}(s)=\sum_{s=1}^{t}R_{0}p_{G}(t+1-s)I(s),$$

where *p*_G_ is a discrete probability distribution for the generation time corresponding to the continuous-time distribution *f*_G_. A natural statistical model for data collected daily or weekly is then to assume that the number of newly infected *I*(*t* + 1), given previous incidence, follows a Poisson distribution with mean parameter as in equation (2.5) (e.g. [3]).

Finally, the quantitative evaluation of many theoretical results requires explicit assumptions about the involved probability distributions and other parameters typical of the disease under study. As illustrations, we have chosen to use Gamma distributions, where possible, because of their flexibility and analytical properties, and parameters compatible with the recent 2014 West Africa Ebola epidemic [3]. Various formulae related to these assumptions are collected in the electronic supplementary material.

## 3. Looking backwards rather than forwards in time

The generation time distribution *f*_G_(*t*) = *β*(*t*)/*R*_0_ describes the variability of the (random) time between the moment of infection of an individual and the moments that this individual infects other individuals. When trying to estimate this distribution from outbreak data, the most common situation is where infected cases are contact-traced, i.e. the infectors of cases are identified, and the duration between the infection times of infector and infectee is ascertained (at least in theory, but see also next section). This seemingly innocent choice of looking backwards rather than forwards in time (measuring duration backwards from an infectee rather than forwards from an infector) actually modifies the distribution of observed times in the early stage of an outbreak when the epidemic grows at an exponential rate (e.g. [17–19]). The reason is that, by looking backwards in time, long generation times will be underrepresented and short generation times will be overrepresented because exponential growth implies that there are many more recently infected individuals who are potential infectors compared to those infected longer ago. As a consequence, if the generation time distribution is estimated from a sample of backward generation times, the resulting distribution *f*_B_(*s*) will be different from the true generation time distribution *f*_G_(*s*).

In fact, it can be shown that the backward generation time has density *f*_B_(*t*) = e^−*rt*^*R*_0_*f*_G_(*t*) (note that equation (2.4) implies that this function integrates to 1). It can also be shown that this density is stochastically smaller than *f*_G_ and thus has smaller mean than *f*_G_ (see the electronic supplementary material). We can in fact say more. One can predict the effect on estimating *R*_0_ of using *f*_B_(*t*) instead of *f*_G_(*t*) assuming that the growth rate *r* is known or approximately known through observations. Since incidence essentially is *R*_0_ × a weighted sum of previous incidence (cf. equation (2.5)) and *f*_B_(*t*) attributes too much weight to recent incidence (shorter generation times), which is higher than earlier incidence, there will be a compensatory underestimation of *R*_0_ (see §7 for illustrations). This effect also takes place using the Euler–Lotka equation (2.4).

If instead *f*_B_(*t*) is used to calculate the exponential growth rate in equation (2.4), assuming that the correct value of *R*_0_ is used, the resulting growth rate *r*_B_ will always be larger than *r*. The exact relation is model specific, but as an example one may consider the simple Markovian SIR model, where the infectious period has an exponential distribution with expected value 1/*γ* and the infectious contacts, in the initial phase of the epidemic, occur with intensity *β* during the infectious period. The resulting *R*_0_ is *β*/*γ*, *r* = *β* − *γ*, *f*_G_(*t*) = *γ* e^−*γt*^ and *f*_B_(*t*) = *β*e^−*βt*^. Then, the resulting *r*_B_ equals *R*_0_ *r*. With typical values of *R*_0_ being between 1.5 and 2, this means that the exponential growth rate will be grossly overestimated (50–100%), when using equation (2.4).

Finally, if an estimate $\;{\hat{\;f}}_{B}(t)$ has been obtained from generation times observed backwards and the exponential increase rate *r* is known or has been estimated from observations, then, in theory, the density *f*_G_(*t*) could simply be estimated as $C\;{\text{e}}^{rt}\;{\hat{\;f}}_{B}(t)$ where *C* is a suitable normalizing constant. However, since this method risks overweighting large observations, it is maybe preferable to do the correction within a suitable parametric form. For instance, if *f*_G_ is a Gamma distribution with parameters *α* and *λ*, then *f*_B_ is again a Gamma distribution, now with parameters *α* and *λ* + *r* (see the electronic supplementary material). By inverting this relation, a suitable correction procedure can be obtained.

The above analysis of the potential bias in ascertaining generation times in the early stage of an outbreak is focused on ‘backward’ contact tracing, because this is the most common situation studied in theory and used in practice, i.e. finding potentially infectious contacts that have occurred in the past. One could, in theory, consider the case of ‘forward’ contact tracing and potential sources of bias in that situation, but many assumptions would be needed for, e.g. how infectivity after discovery would be related to infectivity prior to discovery, for how long the tracing would go on, etc. There is also a theoretical problem, namely that the ‘forward’ generation times do not have the same distribution as ‘backward’ times (e.g. [18]), even in the absence of the exponential growth bias illustrated above.

## 4. Replacing generation times with serial intervals

As described earlier, the generation time is defined as the time between moments of infection of an infector–infectee pair. However, in real life, the infection times are rarely known. Instead, typically, the onset of symptoms is observed. For this reason, the serial interval, which is defined as the time between symptom onsets in the two individuals mentioned above, is often used as a surrogate for the generation time.

We now study the effects of using serial intervals instead of ‘true’ generation times when estimating the generation time distribution $f_{G}(\cdot )$ and on derived quantities, such as *r* and *R*_0_.

**Figure 2.**
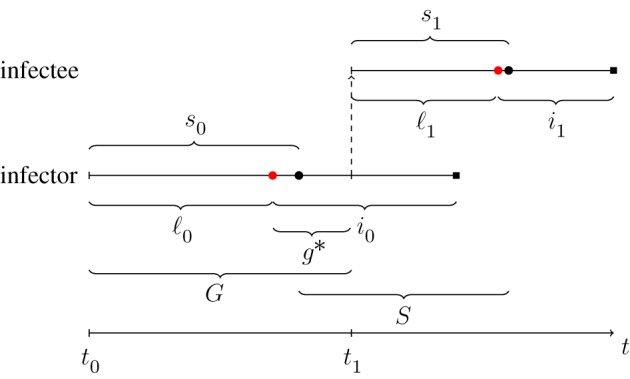
Relationship between generation time *G* and serial interval *S* of an infector and its infectee. The infector is infected at time *t*_0_ and then infects the infectee at *t*_1_. The red circles indicate end of latent period and start of infectious period, the black circles indicate onset of symptoms, and black boxes end of infectious period (either by death or recovery). In the figure, the infectious period starts slightly before onset of symptoms, but, in general, the relationship between these event times is disease-dependent. In the illustration, the serial interval is shorter than the generation time, *S* < *G*, but the opposite relation could equally well happen. (Online version in colour.)

Considering the disease and infectivity history of an individual, starting from the moment of infection, several time periods are of interest (see also figure 2). We denote the time of infection of this individual by *t*_0_, there may be a latent period of length ℓ_0_ until start of infectivity followed by an infectious period of length *i*_0_, and a time from infection to symptoms (incubation period) of length *s*_0_. Assume that another individual is infected by the first one after a time $g_{*}$ within the infectious period *i*_0_, i.e. at time $t_{1}=t_{0}+\ell_{0}+g_{*}$, and that this second individual shows symptoms at time *s*_1_ after infection. Then, the generation time is $G=(t_{0}+\ell_{0}\;+g_{*})-t_{0}$ and the serial interval *S* = (*t*_1_ + *s*_1_) − (*t*_0_ + *s*_0_) (see figure 2 for an illustration).

Although much work has been devoted to estimating the distributions of incubation, latent and infectious periods for various diseases, relatively little has been done regarding their joint distribution. It will be seen below that this joint distribution plays an important role for the relationship between *G* and *S*. Let us only assume, for a start, that the involved times are independent between different individuals and that corresponding periods have identical marginal distributions for different individuals. We may then rewrite the above expressions as

4.1 $$G=s_{0}+(\ell_{0}+g_{*}-s_{0})\;\text{and}\;S=s_{1}+(\ell_{0}+g_{*}-s_{0}).$$

These representations are quite unnatural, but show the common structure of *G* and *S*. For instance, we see that *S* = *G* + (*s*_1_ − *s*_0_) and thus the expected values of *G* and *S* will be equal since *s*_0_ and *s*_1_ are assumed to have identical expected values. We also see in equation (4.1) that *S* is the sum of two independent components (since they regard different individuals) and thus its variance will be the sum of the variances of these components, while the variance of *G* will consist of the sum of the same two variances and a further term, $2\text{Cov}(s_{0},\ell_{0}+g_{*}-s_{0})$, by the rule for variances of sums.

Depending on assumptions, we can now have different results. Under quite usual assumptions of independence between *s*_0_, ℓ_0_ and *i*_0_ and also Var(*s*_0_) > 0, the covariance above will be negative, implying that Var(*S*) > Var(*G*). If we, instead, want the variances of *G* and *S* to be the same, we must require $\text{Cov}(s_{0},\ell_{0}+g_{*}-s_{0})=0$, which is a rather special balance between various parameters in the joint distribution of (*s*_0_, ℓ_0_, *i*_0_) (to simplify matters slightly, one can note that assuming $g_{*}$ to have a uniform distribution in *i*_0_ leads to $\text{Cov}(s_{0},g_{*})=\text{Cov}(s_{0},i_{0})/2$; see the electronic supplementary material). However, it is also theoretically possible to have the reverse relation, e.g. if *s*_0_ = ℓ_0_ and Cov(*s*_0_, *i*_0_) > 0.

One may also investigate whether the distributions of *S* and *G* can be identical, as argued in [3], under the assumption that *s*_0_ = ℓ_0_ (i.e. the end of the latent period/start of infectious period is identical with onset of symptoms). However, since one then has $G=s_{0}+g_{*}$ and $S=s_{1}+g_{*}$, one must impose further conditions (see the electronic supplementary material). The independence of *s*_0_ and $g_{*}$ (or *i*_0_) would be sufficient but not necessary for the result. At least, *s*_0_ must be uncorrelated with $g_{*}$, since variances must coincide. However, we have not been able to find a simple sufficient and necessary condition for equality (see the electronic supplementary material for further details).

The exact relationship between the generation time *G* and serial interval *S* is thus model-dependent, but it always holds that they have the same mean. As for the variances, nothing can be said in complete generality. However, for all existing models we are aware of, it holds that *V*(*S*) ≥ *V*(*G*), with equality requiring rather specific assumptions. So, except in specific cases, the observed serial interval distribution will be a biased estimate of the generation time distribution and will have a larger variance. The quantitative effects of using a distribution with equal mean but larger variance than the true one are again model-dependent, but, for example, assuming Gamma distributions, there will again be underestimation of *R*_0_, given *r*, and overestimation of *r*, given *R*_0_ (see the electronic supplementary material for further details).

A general method to correct the serial interval distribution to obtain an unbiased estimate of the generation time distribution will of course be very model-dependent and necessitate further information about the various stages of disease history. However, within a specific distribution family, such as the Gamma distributions, a correction based only on the ratio between the serial interval and the generation time variances can be implemented (see the electronic supplementary material). The above results show that the elements needed for the correction from Var(*S*) to Var(*G*) are the covariances between incubation period and latent and infectious periods and the variance of the incubation period. These could be estimated from a close observation of some case histories with known time of infection and/or assumptions such as the above-mentioned *s*_0_ = ℓ_0_.

## 5. Multiple exposures

Contact tracing means that reported cases, with known onset of symptoms, are investigated to find out when they have been in contact with infectious individuals, with the aim of finding who the infector was and when the case was infected, thus allowing estimation of the incubation period or, if the symptom onset time of the infector is also known, the serial interval. In practice, when infected individuals are contact traced, certain cases will have one unique possible infection time, but others will have several potential infectors or infection occasions, or no identified exposure. In the first situation, it is clear who the infector was and also how long the incubation period was, and, in the last case, when there is no identified exposure, there is not much to do. But, in the second scenario, it could be any one of the potential exposures that caused the infection, also implying that the incubation period could be one out of several values. In the current section, we describe one way to infer the incubation period distribution of contact traced individuals in this situation, and also to study the effects of not acknowledging the multiple exposures situation. It should be noted that this is not a standard problem in survival data analysis, where it is usually assumed that the time origin of durations is well defined.

Most of the literature about the ‘uncertain origin problem’ in an epidemiological context arose during the 1980s in connection with inference on AIDS data, where the moment of infection of patients was usually not known exactly (e.g. [20–23]). Often, analyses were based on the assumption of a known interval within which infection had occurred and some kind of continuous distribution therein for the moment of infection. The problem reemerged during the SARS pandemic in the early 2000s (e.g. [24,25]), but again with data limited to single exposures during known time intervals. In this paper, we analyse the situation where individuals may have more than one exposure, the times of these exposures are known and where there is no detailed information about the nature/strength of exposures.

Let us start by considering the problem of estimating the incubation period distribution, i.e. the time from infection to symptoms, in the simplest model possible allowing for multiple infection exposures and to formulate an appropriate likelihood. Consider one infected individual with onset of symptoms at time *s* that has been traced for previous infectious contacts and assume that these exposures took place at the time points *e*_1_, …, *e*_*k*_, where *e*_1_ ≤ · · · ≤ *e*_*k*_ < *s*. In order to obtain a likelihood, we introduce some notation and assumptions. Suppose that at time *t*, the rate of infection exposure equals *λ*(*t*), and that the probability of infection upon exposure equals *p* (the same *p* for all contacts whether with the same or different infected individuals; if more detailed contact information is available it would be possible to have different *p*’s for different types of contacts and/or different individuals). Finally, let *g*(*t*) denote the density distribution of the incubation period. For this model, the likelihood for the infected individual with exposures at times *e*_1_, …, *e*_*k*_ and onset of symptoms at *s* is then given by

5.1 $$L(e_{1},\ldots ,e_{k},s)=\left[{\text{e}}^{-\int_{0}^{s}\lambda (u)\;du}\prod_{i=1}^{k}\lambda (e_{i})\right]\times \left[\sum_{i=1}^{k}p{\left(1-p\right)}^{i-1}g(s-e_{i})\right].$$

We will discuss the estimation problem arising from equation (5.1), but we start with some general considerations. It is of course possible to also study more complicated models allowing for individual heterogeneity in susceptibility and/or various types of contacts having different transmission probabilities, both at distinct times and also during extended periods, e.g. household contacts, but here we consider the simplest model still taking multiple exposures into account, compatible with limited data, only number and times of contacts and time of symptoms. Were data to be different, e.g. containing genetic information from whole genome sequencing of the pathogens, different models would be possible (e.g. [26]).

One can imagine several ways to try to avoid the multiple exposures problem. One approach could be to simply assume that the earliest potential infector is the infector, the likelihood contribution related to the incubation time distribution simplifying to *g*(*s* − *e*_1_) (this would approximately be the same as equation (5.1) if *p* ≈ 1). This would however certainly lead to the duration of incubation periods being *overestimated*. The opposite approach, to pretend that the most recent contact was the infector, would similarly lead to *underestimation*. A type of compromise could be to treat all potential contacts as being potential infection times (to different cases). As a consequence, one observation with *k* multiple potential infectors would then result in *k* *independent* incubation periods *s* − *e*_1_, …, *s* − *e*_*k*_, and the likelihood contribution would become $\prod_{i=1}^{k}g(s-e_{i})$. Compared to the likelihood in equation (5.1), where the shorter incubation periods are given relatively lower weight due to the factor (1 − *p*)^*i*−1^, such an analysis would lead to the incubation periods (and serial intervals) being *underestimated* and the precision of the biased estimate overestimated because of the apparent higher number of data points. A related assumption, leading to the same conclusion, would be to treat all potential exposures as equally likely (which would approximately hold true if *p* ≈ 0).

An alternative approach to overcome the difficulty of having multiple potential infectors, is to base inference only on individuals having one exposure, i.e. simply leaving out all contact traced individuals having more than one exposure. This clearly increases uncertainty by using fewer data points. However, it also leads to biased estimates, as we now explain. Individuals having only one exposure and then symptoms must have been infected at this exposure and thus their infection history is certain. However, the fact that no other exposures have happened during the incubation period favours shorter than usual intervals. In fact, the observed time interval will be distributed as the minimum of a typical ‘inter-exposure time’ and a generic incubation time, and will thus have a distribution different from a generic incubation time, leading to underestimation. In order to obtain explicit expressions for the size of the bias, explicit models of the ‘exposure process’ and the incubation time distribution are required.

If we adopt the simple multiple exposure model defined above, it is however possible to estimate the incubation period distribution using the likelihood in equation (5.1). It is reasonable to condition on the number and times of exposure, since these essentially depend on the ‘inter-exposure process’, and to base inference on the second part of the likelihood expression only, containing parameters *p* and the incubation distribution g(⋅). Assuming a parametric form for g(⋅), e.g. a two-parameter gamma distribution, the problem is non-standard but essentially a three-parameter maximum-likelihood problem with natural bounds on parameters. Simulations show that this approach works well if the correct parametric form is adopted (see the electronic supplementary material). It is also possible to find non-parametric (distribution-free) moment estimators of *p*, the mean and the variance of the incubation time at the cost of assumptions about the contact process, e.g. as a constant rate Poisson process. Details about one set of such moment estimators and their performance are given in the electronic supplementary material.

## 6. Counting delayed events

The individual evolution of a disease is often a sequential process of events delayed with respect to some previous event, starting with infection and then followed by, for example, symptoms, notification, admission to treatment, recovery or death, not necessarily passing through all these states nor in that particular order. The prevalence of individuals in some of these disease stages is of public health interest but reliable data may not always be available. During the early phase of an outbreak, information is usually incomplete due to censoring, but also distorted by exponential increase. The estimation problems and possibilities are thus quite different during the initial phase compared to, retrospectively, after or near its end (e.g. [24,27] for some analyses based on more complete data). However, during the exponentially increasing phase of spread, a well-known result from branching process theory (a special case of ‘counting by random characteristics’; e.g. [28]), implies that ratios between counts within a specific time window can be predicted.

Assume that events occur on the time interval [0, *T*]. Assume also that each event may be followed, with a certain probability *p*, by a secondary event after some time having probability density *h*(*s*). Then, assuming that the number of primary events grows exponentially at rate *r*, the fraction *π*(*T*) of secondary events to primary events in [0, *T*] will quickly approach (for *T* not too small)

6.1 $$\pi (\infty )=p\int_{0}^{\infty }{\text{e}}^{-rs}h(s)\;\text{d}s.$$

More details are given in the electronic supplementary material and in §7.5 about the stability of ratios between various events in the simulations.

The above formula illustrates the combined effect of censoring and exponential growth on (theoretical) counts. Thus, taking, for example, infection as primary event and notification as secondary event, knowledge about the notification probability and about the distribution of the time to notification allows estimation of not yet notified cases or of total number of infected from the number of notifications in [0,*T*].

Another useful application of the above result is to count-based estimation of the case fatality rate (CFR). In this case, *p* represents the probability of dying from the disease. The problem has been treated by many authors under many different assumptions of data availability (e.g. [27,29–31]). It is well known that a crude estimator of the type *D*/*N*, *D* denoting number of dead individuals and *N* number of notified individuals in [0, *T*], will underestimate the ‘true’ CFR. In theory, disregarding biased reporting, the underestimation is reflected in the integral part of equation (6.1), which is always less than 1, where *h* now denotes the probability density function of the time between notification and death. Consequently, knowledge of *r* and of the distribution *h* could be used to correct the naive estimate *D*/*N*. As an illustration, if the distribution *h* is assumed to be a simple exponential distribution with expected value *μ*, the correction factor would simply be 1 + *rμ*.

In [3], another approach is used, namely estimating only on cases with a known final destiny (death or recovery) within [0, *T*]. Let us denote by *N* those notified in [0, *T*] and, among those, by *R* the number of cases who are observed as recovering and, as before, by *D* those that have died. Then, another application of equation (6.1) gives that the fraction *R*/*N* will be close to (1 − *p*)*ρ*, where *ρ* is the integral part of equation (6.1) calculated with the probability density of the time from notification to recovery. As before, the fraction *D*/*N* will be close to *pδ*, where *δ* is the corresponding expression involving the probability density function of the time from notification to death, and thus the estimator *D*/(*D* + *R*) will be close to

$$\frac{\;p\delta }{p\delta +(1-p)\rho }=\frac{\;p}{p+(1-p)\frac{\rho }{\delta }}.$$

Thus, the estimate will be (approximately) unbiased only if *ρ* = *δ*. If *ρ* < *δ*, then the CFR will be overestimated. This happens if the time to remission is stochastically larger than the time to death, which is the case for many diseases; for instance, this seems to be the case for Ebola (see §7.4). However, the reverse case, i.e. *ρ* > *δ*, is also interesting, e.g. for influenza [29]. Of course, this analysis only considers the theoretical effects of censoring and exponential growth, not other effects such as differential reporting, reporting delays or general underreporting.

## 7. Results

We now illustrate the numerical consequences of our findings based on realistic parameter values and model based formulae and simulations. Mostly, we have chosen parameter values compatible with the recent Ebola epidemic in West Africa as described in [3]. Details about theoretical derivations and about the simulation program and related results are reported in the electronic supplementary material.

### 7.1. Looking backwards

We assume that the generation time follows a gamma distribution *G* ∼ *Γ*(*α*, *λ*) with (*α*, *λ*) = (3, 0.2), implying a mean of 15 days and standard deviation 8.66 days, and that *R*_0_ = 1.7. This induces a true exponential growth rate *r* = 0.0387 (per day). The generation time when looking backwards in time will also follow a gamma distribution, but with different parameters and mean = 12.6 days and standard deviation = 7.26 days. If the true value *r* = 0.0387 is used in equation (2.4) in conjunction with the backward generation time distribution, the result is $R_{0}^{\left(B\right)}=1.57$ as compared to the true value *R*_0_ = 1.7, an 8% underestimate. If the exponential growth rate is computed for this (backward) generation time distribution and the true *R*_0_, the induced exponential growth rate then equals *r*_B_ = 0.0462. Thus, the growth rate is overestimated by 19%.

### 7.2. Serial intervals

We illustrate the consequences of overestimating the variance of the generation time distribution by using serial intervals instead of generation time data in the simplified framework where both distributions are of the Gamma type and the difference is represented by the coefficient of variation of the serial interval distribution being larger than that of the generation time distribution by a factor *c* > 1, while the means are equal, as predicted by theory (see §4). If we assume the same basic parameter values as in the preceding subsection (i.e. the generation time follows a gamma distribution *G* ∼ *Γ*(*α*, *λ*) with (*α*, *λ*) = (3, 0.2) and *R*_0_ = 1.7), and calculate the biases resulting from, for example, *c* = 1.1, 1.2, 1.5 and 2, we find that the corresponding *R*_0_ values, assuming the true *r* value is used in equation (2.4), are underestimated by 0.9, 1.8, 4.8 and 9.6%, respectively, while the corresponding *r* values, assuming *R*_0_ = 1.7, are overestimated by 1.9, 4.1, 12.3 and 32.9%, respectively. Thus, sizeable bias can be obtained if the serial intervals are much more variable than the generation times.

In [3], the generation time distribution was estimated from observed serial intervals, under the assumption that the distributions would be equal, assumed to be a consequence of the exact coincidence of onset of symptoms and beginning of infectious period. Under this assumption, there is of course no bias effect due to increased variance. However, it is interesting to note that the simulation results (see the electronic supplementary material) show that the serial intervals suffer the same distortion as generation times, due to exponential growth.

### 7.3. Multiple exposures

In order to estimate the effects of basing the estimates of durations on individuals having only one exposure (see §5), some additional assumptions are needed. For the recent Ebola epidemic, it is found [3] that the incubation period distribution *f*_*D*_, assumed to be equal to that of the latent period, is Gamma distributed with mean 11.4 days and s.d. = 8.1 days and that generation times are Gamma distributed with mean 15.3 days and s.d. = 9.3 days. It is also reported that approximately 25% of the contact traced individuals had one unique infector and 75% had more than one potential infector. We will use the above parameter values for this example. With the complete data, it would have been possible to estimate the contact rate *λ* and the probability *p* to get infected by a close Ebola contact separately. Here we can only use that 25% had a single contact. We simply assume that *p* = 0.5 and equate $P(\text{a single contact})=p\int_{0}^{\infty }{\text{e}}^{-\lambda s}f_{D}(s)\;\text{d}s$ to the empirical value 0.25. The result is that *λ* = 0.0725 per day (so about one close contact every two weeks for the contact-traced individuals). Once values for *p* and *λ* are available, one can compute the mean incubation period for observations having only one possible infector:

$$\begin{array}{rl} & E(D\;|\text{one possible infector})\\ & =\int_{0}^{\infty }spf_{D}(s)\;{\text{e}}^{-\lambda s}\;\text{d}s/P(\text{one possible infector})\approx 8.1. \end{array}$$

Thus, the mean incubation period for infectees with only one potential infector will be 11.4 − 8.1 = 3.3 days shorter than the mean incubation period had all observations been used. This in turn implies that the mean generation time from the same data would be underestimated by 3.3 days, giving a mean of 12 instead of 15.3 days. Assuming that the standard deviation remains unchanged (= 9.3 days), the estimated generation time distribution would be Gamma distributed with mean 12 and s.d. = 9.3 days. Using this generation time distribution, instead of the true one, in equation (2.4), assuming *r* = 0.0383 to be known (e.g. estimated from the observed growth rate), leads to *R*_0single_ = 1.50, an underestimation of 12%. Conversely, assuming *R*_0_ = 1.7, the exponential growth rate estimated from the contact traced individuals having only a single unique infector would approximately equal *r*_single_ = 0.0522, which overestimates the true value by 36%.

Instead, to study the performance of maximum-likelihood estimation based on equation (5.1) and on an alternative set of moment estimators, we have simulated observations from 500 individuals (see the electronic supplementary material for details), showing that estimates of *p* and the parameters of the incubation period distribution seem reasonably unbiased, given the parameter setting and assuming the correct distributional form in the likelihood method. If the incubation period has a distribution differing from the assumed (gamma) model distribution (the log-normal distribution, in our simulation), the moment-estimators still perform well, but the maximum-likelihood estimates of mean and variance derived under the assumption of gamma distributed incubation times now acquire some bias. The speed of convergence of estimates and further properties under misspecification of assumptions need further study, but this initial experiment shows that unbiased estimation based on all observations is possible.

### 7.4. Case fatality rate example

The WHO Ebola Response Team [3] report that the average time from symptoms to death is 5 + 4 = 9 days, while to remission the average time is 5 + 12 = 17 days. The numerical consequences of the results in §6 can be sizeable. If we assume that we have exponential growth with a doubling time of say 20 days, the growth rate *r* becomes 0.0347. Under the simplifying assumption that the time from notification to death follows an exponential distribution with mean *μ* = 9 days, the multiplier 1/(1 + *rμ*) becomes 0.76, i.e. there is an underestimation of about 24% of the CFR, using the simple estimator *D*/*N*. However, similarly the factor *ρ* will be 0.63 and, assuming a CFR of 70%, say, the estimator relying only on cases with a known final destiny will overestimate the CFR by approximately 5% of its value.

### 7.5. A simulation study

In order to better study the behaviour of various observables during the early phase of an outbreak, we have conducted simulations of the basic epidemic model and evaluated various statistics. Parameter values were chosen to be similar to the recent Ebola epidemic in West Africa. Some of the results have already been commented upon in the preceding sections, but further results about the time it takes to reach predefined levels of notified cases, the stability of ratios between numbers of individuals in different disease stages, the relationship between generation times and serial intervals and predicting the size of the epidemic at a later time as well as details about the simulation and its parameters are presented in the electronic supplementary material.

## 8. Discussion

In this paper we have, by means of modelling, analysis and heuristics, both theoretical and simulation-based, studied inferential problems in an ongoing epidemic outbreak in its early stage. Our analyses give insights into where biases might ‘hide’ and also how to avoid these biases. We have studied three potential sources of bias: (1) backward estimation of generation times (contact tracing), (2) using serial intervals instead of generation times and (3) contact tracing leading to several potential infectors thus making the time of infection uncertain. Importantly, all three sources lead to biases in the same direction, causing the basic reproduction number *R*_0_ to be *underestimated* if the epidemic growth rate *r* is correctly estimated. The converse is also true, namely that the growth rate will be *overestimated* if a correct estimate of *R*_0_ is available, but this situation is likely to be less common in practice.

The biases have been numerically evaluated, as illustrations, in a setting resembling the recent 2014 Ebola epidemic. To evaluate the combined biasing effect in this setting is a complicated exercise, depending on exactly how estimation is performed and presented but, assuming independence (i.e. multiplicative action), the total effect could be as large as, for example, 23% underestimation of *R*_0_ (assuming a 50% increase in the coefficient of variation of the serial interval). In other settings, the effects could be quite different. In the main sections and in the corresponding sections of the electronic supplementary material, general formulae, if possible, or at least formulae under assumptions of Gamma distributions, if results depend on assumed distributions, are available and applicable in other parameter settings. It should be noted that even moderate errors in *R*_0_, especially if the true value is not very large, can have large consequences. As numerical illustration, assume that the true value of *R*_0_ is 1.7, but that the estimate is negatively biased by 10% or even 23%, depending on how estimation is performed. Then the biased estimate would be around 1.53 or 1.31, respectively. Such a difference can have quite large consequences when planning control measures. For instance, the critical immunization level (both for vaccination and any other measure aimed at reducing infection) is usually calculated as *v*_*c*_ = 1 − 1/*R*_0_. For the true *R*_0_, this results in $v_{c}=41\text{\%}$, while the lower biased estimates yield ${\hat{v}}_{c}=35\text{\%}$ or 24%, respectively. The underestimation of *R*_0_ may hence lead to suggested preventive measures that are insufficient to stop the spread.

The focus of the paper has been on studying potential biasing effects originating from a typical set of observables in the initial phase of an outbreak. However, there are also some positive observations. The stability of proportions of individuals in different phases of disease during the increasing phase is one, since quite good estimates of the total number of infected or not yet notified infected could be made, based on number of dead patients or notified ones, if good information about the related stage duration distributions is available. Another positive observation is that accurate inference in the multiple infector problem seems possible, although more research is needed. Finally, many biases can be understood and corrected for if the sampling situation is correctly modelled. It may be difficult to obtain simple analytical results, but simulation can then reveal the performance of various estimation procedures.

In the paper, it was assumed that incidence was reported on aggregated level without additional information on household or spatial structure of the reported cases. If more detailed data were available, then more sophisticated models taking such heterogeneities into account may be used. This will improve the statistical analyses in that the model better describes the spreading patterns of the disease. The biases under focus in the present paper will however remain, but it is an open problem to study if they are reduced or increased when compared with the present homogeneous modelling assumptions. The results in this paper also indicate what other types of data would have been useful to avoid or decrease the discussed biases. For instance, more detailed observation of the joint distribution, or at least first and second order moments, of the various phases of the disease history (incubation, latency, infectiousness, etc.) would have improved the conversion of serial intervals to generation times; genotyping of the infectious agent in infected individuals may improve inference about infection chains and thus avoid the multiple infector problem and also allow direct estimation of *R*_0_ in infection trees.

Of course, there are also many other problems related to data from an emerging outbreak not treated in the current paper, important ones being underreporting, selective reporting and reporting delays, but also batch-reporting of numbers. A rather different type of potential source of bias, also not studied here, is when model assumptions are violated. For example, it has been assumed that there was no individual or society-induced changing behaviour during the data collection period, and social or spatial effects on spreading patterns have been ignored. Social structures have been shown to have limited effects for estimation in emerging epidemics [2], but spatial effects [32] clearly play a role in disease spread, and their effect on parameter estimates is yet to be investigated. Changing behaviour probably kicks in early in emerging outbreaks of serious diseases like Ebola, and are hence also important to include in future inferential procedures for emerging epidemic outbreaks.

Still, it is our hope that the results can help in improving future analyses of emerging outbreaks and the important efforts to guide health authorities in predictions and identifying possible preventive measures.

## Supplementary Material

Supplementary_Information

## Supplementary Material

simulation.c

## Supplementary Material

multiple_infectors.R

## Data accessibility

Details of proofs and simulation results are available in the file Supplementary Information.pdf, the code (in C) for outbreak simulation in the file simulation.c and the code for simulating multiple infectors data (in R) in the file multiple infectors.R, all available as electronic supplementary material to this paper.
