# Supplementary material for: Estimation in emerging epidemics: biases and remedies
Source: J R Soc Interface. 2019 Jan 16;16(150):20180670. doi: 10.1098/rsif.2018.0670 (PMC6364646; doi:10.1098/rsif.2018.0670)
Supplement: Supplementary_Information [file rsif20180670supp1.pdf]

Estimation in emerging epidemics: biases and remedies
Supplementary Information (SI)

Tom Britton*and Gianpaolo Scalia Tomba†‡

December 13, 2018

Contents

1 The simulation model and some interesting results **2**

2 Delayed observations **4**

3 "Backward" observation of generation times **4**

4 The E-L equation and the Gamma distribution **6**

5 Multiple exposures and estimating the incubation period **7**

6 Generation times and serial intervals **8**

7 Estimating the growth rate **9**

8 References **11**

Notation

In what follows, frequent reference is made to Gamma distributed quantities. We use the notation $\Gamma(\alpha, \lambda)$ for a Gamma distribution with shape parameter α and scale parameter λ, having expected value α/λ and variance α/λ^2. It should also be noted that, in the $\Gamma(\alpha, \lambda)$ distribution, the coefficient of variation (ratio of standard deviation to mean) is $1/\sqrt{\alpha}$ and that, given the expected value μ and the variance σ^2, one has $\alpha = \mu^2/\sigma^2$ and $\lambda = \mu/\sigma^2$.

*Dept. of Mathematics, Stockholm University, 10691 Stockholm, Sweden, <tomb@math.su.se>.
†Dept. of Mathematics, Univ. of Rome Tor Vergata, 00133 Rome, Italy, <scaliato@mat.uniroma2.it>.
‡Corresponding author

Code

Two sections contain the computer codes used to simulate the branching process representing the early phase of an outbreak with possibilities to trace infection chains (written in Standard C) and to simulate the situation described in the multiple infectors section, with possibilities to collect statistics of various kinds (written in R). The codes come with no guarantee of correctness (but, of course, we hope that they are correct :-) and may of course be changed to have different output or parameters. Maybe they can even be improved for specific applications...

1 The simulation model and some interesting results

Model structure The simulated epidemic has been constructed to be close to the parameters reported for the recent Ebola epidemic by the WHO Ebola Response Team [1], with the exception of the incubation period which has been allowed to be slightly different from the latent period. Each infected individual follows a stochastic SEIR model with all time periods following Gamma distributions, the time unit being 1 day. The latent period E is assumed to be $\Gamma(2, 1/5)$ ($mean = 10$, $sd = 7.1$) and the infectious period $\Gamma(1, 1/5)$ ($mean = 5$, $sd = 5$). After the infectious period, the individual may either recover, with probability 30%, or die, with probability 70%. The time until recovery is assumed to be $\Gamma(4, 1/3)$ ($mean = 12$, $sd = 6$) and the time to death $\Gamma(4/9, 1/9)$ ($mean = 4$, $sd = 6$). Furthermore, each individual has an incubation period (time until symptoms) which is assumed to be similar to the latent period, but with some variation. The incubation period is given by E times a uniformly distributed variable in the interval [0.8,1.2]. It is assumed that a case is reported when symptoms arise. Finally, during the infectious period, new cases are produced with rate 0.34/day, resulting in $R_0 = 0.34 \cdot 5 = 1.7$.

The stochastic process as such is a Crump-Mode-Jagers branching process, in which the expected incidence of infections $b(t)$ satisfies (see [2]) the renewal equation

$$b(t) = R_0 \int_0^t b(t-u) f_G(u) du + R_0 f_G(t).$$

The solution to this equation quickly approaches exponential growth $\sim Ce^{rt}$, where r is the so called Malhtusian parameter. The same exponential growth, although with different constants, will also apply to the total number of infected, reported, recovered, dead, etc., individuals.

It is easily shown (see e.g. [3]) that the generation time distribution f_G in the above model is $\Gamma(3, 1/5)$ ($mean = 15$, $sd = 8.7$) and that the Malthusian parameter is $r = \lambda(R_0^{1/\alpha} - 1) = 0.0387$. The deterministic doubling time is 17.9 days.

Duration of simulations Only "exploding" trajectories, corresponding to "big" outbreaks, are kept. Outbreaks start with 1 infected individual and are rejected if they do not reach 4500 reported cases. At the time of reaching this level, some statistics are collected and then the simulation is continued for 6 weeks further. The purpose of this continuation is that a prediction of the final level 6 weeks later is attempted, based on

the first 4500 cases. Statistics are based on 1000 accepted trajectories.

Programming details The simulation program was written in standard C, because of the need to keep links (pointers) between infectors and infectees, in order to simulate contact tracing, and executed on a desktop computer. Results from the simulations were elaborated using the software R to produce the R_0 and r estimates, and the 6 week projections as well as statistical summaries. Random number generation for Gamma distributions with non-integer shape parameter used the algorithm of Phillips & Beightler [4].

Time from introduction of first case to 4500 notified Average and median approximately 200 days ($sd = 33$), but with a range of $[123, 358]$. A deterministic estimate would probably be $\ln(4500)/r = 217$, where r is the exponential increase rate. The slight difference might be due to the conditioning on non-extinct trajectories. Furthermore, the largest part of variability is in the first part of the epidemic. If one divides the time period in a first part until the first 100 (cumulative) cases are reached and a second part until level 4500 is reached, one finds that the first part has average length 102 days with $sd = 28$ and the 95% central range $[63, 174]$ and that the second part has mean 98 days, $sd = 10$ and 95% range $[83, 119]$.

Stability of various ratios when 4500 cases have been notified At the time of 4500 cases notified (in total), the numbers of individuals infected, who had died or who had gotten well, were recorded. It should be noted that the total number of infected is not realistically observable, but of interest to estimate. It should also be noted that all numbers above are examples of delayed observations, from infection to notification and from notification to final destiny. One should therefore expect that the ratios should stabilize around values given by the formula (8) in Section 6 of the main paper. The ratio notified/infected was, on average, 0.70 with 95% of values between 0.68 and 0.72, essentially identical to the theoretical prediction based on "knowing" the incubation time distribution, which was assumed to also be the time from infection to notification. The narrow range of the observed ratios indicates that this is a viable method to predict the true actual size of the outbreak from the number of notified cases, given that good estimates of the distribution of time to notification and exponential increase rate are available. An analogous result holds for the ratios of infected to dead or recovered individuals. With the specific parameters of the simulations, at 4500 notifications, on average 3350 of them had either recovered or died, and the remaining 1150 remained between notification and final destiny. At the time of 4500 notifications, an average of 1900 additional individuals had been infected but not yet notified.

Observed generation times and serial intervals In each simulation run, 500 generation times and 500 serial intervals were sampled from the first 4500 notified individuals by systematically taking every ninth individual until the sample was complete. The times and intervals were ascertained "backwards", i.e. the infector of the chosen individual was identified and time distance between the respective infection or symptom times was recorded. The distributions of sample means are reasonably concentrated around the respective central values, 12.5 for serial intervals and 12.6 for generation times. It should be remembered that theory predicts that both should have the same expected value which

should be less than the true expected generation time, which is 15. Theory again predicts that the backward generation time should have mean 12.57, which is not far from what is observed. The variance of the true generation time is 75 ($sd = 8.7$) and both variance estimates from the simulation samples tend to be much less, somewhat above 50 ($sd = 7.1$). This also leads to the useful conclusion that serial intervals are affected by the same "contraction" as generation times when ascertained "backwards", at least in the chosen parameter setting.

Predicting the size of the outbreak at a later time Finally, we study the performance of the renewal equation (Section 2, Eq. (5), main paper) approach proposed in [1]. This approach is intended to allow estimation of R_t (in our simulation, R_t is kept constant $= R_0$ all the time and the method is adapted accordingly) and to further allow prediction of cases 6 weeks (42 days) after the last observed datum. The results, using probabilities derived from observing backward serial intervals, thus biased with respect to the true generation time distribution, indicate that this method seriously underestimates R_0 but has good predictive power anyway. The 95% range of the ratio predicted/true values at 6 weeks after the level 4500 notified has been reached is $[-10\%, +12\%]$, which is slightly better than just using a growth rate based estimate (i.e. just multiplying with $\exp(42r)$, using some good estimate of the growth rate r) derived from the first 4500 cases. This is perhaps natural, since the method amounts to an adaptive regression method which has fitted all the observed data up to level 4500 as well as possible, and then extrapolated this fit. As predicted by theory, the estimate of R_0 that results from this method underestimates the true value, in fact the true value 1.7 is never reached in 1000 simulations. The average estimate of R_0 is 1.57, with 95% range $[1.51, 1.63]$, compared to the true value 1.7, thus having an average bias of -8%.

2 Delayed observations

Suppose that events occur with expected intensity $\lambda(s)$ (cumulative intensity $\Lambda(s)$) on the time interval $[0, T]$. Assume also that each event is observed after a delay which is distributed according to the density $h(s)$ with cdf $H(s)$. Then the expected number of observed events on [0,T] is

$$\int_0^T \lambda(s)H(T-s)ds$$

which constitutes the fraction

$$\pi(T) = \int_0^T \frac{\lambda(s)H(T-s)}{\Lambda(T)}ds$$

of the expected number of events on the interval. If the intensity $\lambda(s)$ is constant or even polynomially growing, it can be shown that $\pi(T) \to 1$ as T grows large, while, if the intensity grows exponentially, i.e. $\lambda(s) \sim e^{rs}$, then this fraction quickly approaches (after some simple integration steps)

$$\pi(\infty) = r\int_0^\infty e^{-rs}H(s)ds = \int_0^\infty e^{-rs}h(s)ds$$

as T grows large, which is equivalent to calculating the expected value $E(e^{-rD})$, with D having a probability distribution with density h. If D has distribution $\Gamma(\alpha, \lambda)$, then $\pi(\infty) = (\frac{1}{1+rE(D)/\alpha})^{\alpha}$. It is interesting to note that this is a decreasing function of α, for fixed r and $E(D)$. Thus the case $\alpha = 1$, i.e. the Exponential distribution, yields the largest possible value among Gamma distributions with given mean.

3 "Backward" observation of generation times

Observing generation times, i.e. the time between infection of one individual and another one infected by the first one, has been discussed by several authors, e.g. [3,5,6]. In the exponentially increasing phase of a homogeneously mixing model, the distribution of times observed "backwards", starting from a randomly chosen newly infected individual, will have density $f_B(t) = e^{-r_G t} R_0 f_G(t)$, where f_G denotes the generation time distribution, R_0 the basic reproduction number of the disease and r_G the related Malthusian parameter (this result is approximate in the sense that the truncation effect of the time origin is disregarded). The parameter r_G satisfies the Euler-Lotka (E-L) equation

$$1 = \int_0^\infty e^{-r_G s} R_0 f_G(s) ds$$

If one solves the E-L equation for the Malthusian parameter r_B using the density $f_B(t)$ instead of $f_G(t)$, but with the true R_0, one obtains

$$1 = \int_0^\infty e^{-r_B s} R_0 f_B(s) ds = \int_0^\infty e^{-r_B s} R_0^2 e^{-r_G s} f_G(s) ds.$$

This equation can be rewritten as

$$1/R_0^2 = E(e^{-r_B T} e^{-r_G T})$$

where T has the generation time distribution f_G. Because both functions of T in the expectation are monotone decreasing, their covariance is positive and thus

$$1/R_0^2 = E(e^{-r_B T} e^{-r_G T}) \geq E(e^{-r_B T}) E(e^{-r_G T}) = E(e^{-r_B T}) \frac{1}{R_0}.$$

Since $E(e^{-rT})$ is a decreasing function of r and $E(e^{-r_G T}) = 1/R_0$, and since the above inequality translates to $E(e^{-r_B T}) \leq 1/R_0$, we have that $r_B \geq r_G$.

Conversely, one may think of solving for the basic reproduction number R_B using the backward generation time distribution f_G but the correct exponential increase rate r_G in the E-L equation. Thus

$$1 = \int_0^\infty e^{-r_G s} R_B e^{-r_G s} R_G f_G(s) ds \geq \frac{R_B}{R_G} \int_0^\infty e^{-r_G s} f_G(s) ds \int_0^\infty e^{-r_G s} f_G(s) ds = \frac{R_B}{R_G} \quad ,$$

again using the covariance argument. Thus $R_B \leq R_G$

By the same kind of argument, denoting a time with distribution f_B by T_B and one with distribution f_G by T_G, one can show that, in general, T_B is stochastically smaller than T_G, in the sense that

$$P(T_B \leq a) \geq P(T_G \leq a), \forall a \geq 0.$$

This is verified by rewriting the wanted conclusion as

$$\int_0^a e^{-rt} R_0 f_G(t)dt \geq \int_0^a f_g(t)dt$$

and, noting that $R_0 = \left(\int_0^\infty e^{-rt} f_g(t)dt\right)^{-1}$, as

$$\int_0^a e^{-rt} R_0 f_G(t)dt \geq \int_0^\infty e^{-rt} f_g(t)dt \int_0^a f_g(t)dt,$$

which can be restated as (expectations according to f_G)

$$E\left(e^{-rT} 1_{\{T\leq a\}}\right) \geq E\left(e^{-rT}\right) E\left(1_{\{T\leq a\}}\right).$$

which is true, again because the two function on the LHS are both non-increasing in T. This ordering, for instance, implies that $E(T_B) \leq E(T_G)$.

4 The E-L equation and the Gamma distribution

If the probability density f in the E-L equation, written in the form

$$1 = \int_0^\infty e^{-rs} R f(s) ds$$

is a $\Gamma(\alpha, \lambda)$ distribution, the relations in the preceding section can be explicited. The equation becomes

$$\frac{\lambda^\alpha}{(\lambda + r)^\alpha} = \frac{1}{R}$$

and thus $r = \lambda(R^{1/\alpha} - 1)$ or $R = (1 + r/\lambda)^\alpha$.

Thus, if the parameters of the Gamma distribution are known (alternatively, mean and variance), the exponential growth rate can be calculated if R_0 is known or, viceversa, R_0 calculated if the exponential growth rate r is known.

If the generation time distribution is $\Gamma(\alpha, \lambda)$ and, denoting the corresponding R_0 and r-values by R_G and r_G, the "backwards" generation time distribution will be $\Gamma(\alpha, \lambda + r_G)$ (see [3]). If this density is used to solve for the exponential growth rate $r = r_B$, assuming R_G as R-value, or solving for $R = R_B$, assuming r_G as r-value, in the general E-L equation above, then, after some simplification,

$$r_B = R_G^{1/\alpha} r_G$$

and

$$R_B = \left(1 - \left(\frac{r_G}{\lambda + r_G}\right)^2\right)^\alpha R_G.$$

Finally, if the generation time G has a $\Gamma(\alpha, \lambda)$ distribution and another time S has a Gamma distribution with the same mean but larger variance so that the coefficient of variation of S is larger, by a factor $c > 1$, than the coefficient of variation of G, S will have a $\Gamma(\alpha/c^2, \lambda/c^2)$ distribution (this situation relates to the problem treated in Sections

4 and 7.2 in the main paper). Then, straightforward calculations applied to using this density to solve for the exponential growth rate $r = r_S$, assuming R_G as R-value, or solving for $R = R_S$, assuming r_G as r-value, in the general E-L equation above, yields, after some simplification,

$$r_S = \frac{1}{c^2} \frac{R_G^{c^2/\alpha} - 1}{R_G^{1/\alpha} - 1} r_G$$

and

$$R_S = \frac{\left(1 + \frac{r_G}{\lambda} c^2\right)^{\alpha/c^2}}{\left(1 + \frac{r_G}{\lambda}\right)^{\alpha}} R_G$$

It is again straightforward to show that, all other parameters fixed, r_S is an increasing function of c for $c \geq 1$ and that R_S, all other parameters fixed, is a decreasing function of c for $c \geq 1$. Thus, use of a Gamma distribution with larger variance than the generation time distribution but same mean always leads to overestimation of the exponential growth rate and underestimation of the basic reproduction number, given the true value of the other parameter, since the value $c = 1$ corresponds to the true value generated by the generation time distribution.

5 Multiple exposures and estimating the incubation period

As discussed in Section 5 of the main paper, in order to estimate the parameters of the incubation time distribution and the infection probability per contact p, once assigned a parametric model g with parameters θ, it should be possible to use maximum-likelihood techniques on the relevant part of the model likelihood, conditioning on the number k and times $\{e_i\}_1^k$, of exposure, with s being the time of symptoms,

$$L = L(\theta, p) = \sum_{i=1}^{k} p(1-p)^{i-1} g(s - e_i).$$

It is also possible to find moment relations that allow estimation of p and mean and variance of the incubation time distribution, under some further assumptions on the exposure process.

Suppose that the exposure process is modelled as a Poisson process with constant rate μ with $t = 0$ $(= e_1)$ at the first contact with an infective, that the probability of infection per contact is p, independently at each contact, and that the time from infection to symptoms is denoted by T, with mean $E(T)$ and variance $Var(T)$.

Then the index I of the contact that infects will be Geom(p), i.e. $P(I = k) = p(1 - p)^{k-1}, k = 1, \ldots$. After that, the number of contacts S before symptoms appear will have a Poisson distribution with mean μT, given T. The number of observed contacts will be $C = I + S$, with summands independent. The relations

$$E(C) = 1/p + \mu E(T)$$

Table 1: Empirical 95% central intervals of parameter estimate distributions from 1000 simulated samples of 500 individuals with true values $p = 0.5$, $E(T) = 11.4$ and $s.d.(T) = 8.1$. Simulations from two situations, T being gamma-distributed or log-normal, and ML-estimation based on gamma-distribution or Moment estimation being used.

Model,estimator	p (=0.5)	$E(T)$ (=11.4)	$s.d.(T)$ (=8.1)
$T \sim \Gamma$, ML	(0.500, 0.503)	(11.41, 11.47)	(8.11, 8.18)
$T \sim \Gamma$, Mom	(0.503, 0.508)	(11.39, 11.63)	(7.57, 8.22)
$T \sim LogN$, ML	(0.486, 0.488)	(10.80, 10.85)	(6.33, 6.39)
$T \sim LogN$, Mom	(0.502, 0.507)	(11.36, 11.61)	(7.52, 8.17)

and

$$Var(C) = (1-p)/p^2 + \mu E(T) + \mu^2 Var(T)$$

will then hold.

Denote by S the time from first contact to symptoms. Then S is the sum of the time until infection and the incubation time. The time until infection is, given I, Gamma distributed with parameters $I-1$ and μ. Thus

$$E(S) = \frac{1-p}{p}\frac{1}{\mu} + E(T)$$

and

$$Var(S) = \frac{1-p}{p}\frac{1}{\mu^2} + \frac{1-p}{p^2}\frac{1}{\mu^2} + Var(T).$$

Since $E(C)$, $Var(C)$, $E(S)$ and $Var(S)$ can be estimated from data, the four equations can be used for moment estimation of p, μ, $E(T)$ and $Var(T)$.

Both ML-estimation and moment estimation have been implemented in a small simulation experiment, with 1000 replicates of estimation based on 500 individuals, each having a number of contacts C and a time S between first contact and symptoms. In the simulation, it was assumed that $p = 1/2$, that the incubation period had mean 11.4 ($sd = 8.1$) and that contacts happened according to a Poisson process with intensity 0.0725 (mean interval between contacts = 13.8 days). To test the stability of the estimation methods, one simulation run was performed with Gamma-distributed incubation times, assuming that the target distribution in the ML-method was effectively Gamma and another run using log-normally distributed times with the same mean and variance but still using the Gamma distribution as target in the ML-estimation.

From the simulation results, shown in Table 1 one may conclude that the ML-method works well if the correct distribution is assumed, but less well in case of misspecification, while the moment method seems quite stable, maybe with a hint at downward bias with the lognormally distributed data. However, further research is needed about the best moment expressions to use and possible other approaches to this estimation problem.

6 Generation times and serial intervals

About the distributions of G and S In Section 4 of the main paper, the possibility that the distributions of generation times G and serial intervals S be identical is investigated.

According to the representations in that Section, if the end of the latent period/start of infectious period exactly coincides with the appearance of symptoms ($s_0 = \ell_0$), one then has $G = s_0 + g_*$ and $S = s_1 + g_*$, where s_0 and s_1 are the latent periods of infector and infectee, respectively, assumed independent and identically distributed, and g_* is a part of the infectious period i_0 of the infector, thus potentially dependent on s_0. It is then evident that independence of s_0 and g_* (or i_0, depending on how g_* is constructed) implies equality of the distributions of G and S. The (theoretical) question remains, however, if this condition is also necessary. If it is not, a presumably weaker sufficient condition would suffice. In the abstract, the question can be answered: it is possible to have three random variables (A, B, C) (representing respectively s_0, s_1 and g_*) such that A is independent of B and B is independent of C and the distributions of A+C and B+C are the same, but A and C are not independent. The probably simplest construction showing this is a distribution on the "cube" $\{0, 1, 2\}^3$ (with points denoted by triplets of the form abc, where a,b and c can range in $\{0, 1, 2\}$). Assign probability 0 to points {001,011,021,102,112,122,200,210,220}, probability 1/27 to points {000,010,020,101,111,121,202,212,222} and finally probability 2/27 to the remaining points {002,012,022,100,110,120,201,211,221}. Then it is easily checked that the marginal distributions of A, B and C are all (1/3,1/3,1/3) on $\{0, 1, 2\}$, that the marginal distributions of A+C and B+C are both (3/27, 6/27, 9/27, 6/27, 3/27) on $\{0, 1, 2, 3, 4\}$ and, finally, that A is independent of B and B independent of C but that A is not independent of C.

In Section 4 of the main paper, it is shown that $\text{Cov}(s_0, \ell_0 + g_* - s_0) = 0$, which under the assumption $s_0 = \ell_0$ reduces to $\text{Cov}(s_0, g_*) = 0$, is necessary for the distributions of G and S to be equal, but we have not been able to formulate a necessary and sufficient condition.

On the simulation model and results In Section 4 of the main paper it is shown that $E(G) = E(S)$ but that $Var(S) = Var(G) + 2Cov(s_0, \ell_0 + g^* - s_0)$ (it should be noted that the distribution of the infectious period and thus of g^* depends on a size-biased version of the model infectious period (see e.g. [5]), but that this does not affect the present argument). Since the simulation model assumes independence between latent period ℓ_0 and the infectious period, upon which g^* depends and that $s_0 = u\ell_0$, where $u \sim \text{Uniform}[0.8, 1.2]$, the difference between $Var(S)$ and $Var(G)$ can be written as $2(Cov(u\ell_0, \ell_0) - Var(u\ell_0))$. Since $E(u) = 1$, this reduces to $2E(\ell_0^2)(1 - E(u^2))$. Since $E(\ell_0^2) = 150$ and $E(u^2) = 1.0133$, we should have $Var(S) - Var(G) = 3.99$. Furthermore, in the model we have $G \sim \Gamma(3, 1/5)$ and thus $Var(G) = 75$. In the simulation results, we find the estimates $Var(G) \approx 52.4$ and $Var(S) \approx 54.6$. However, the generation times are observed "backwards", in which case theory predicts that the observed distribution should change from $\Gamma(3, 0.2)$ to $\Gamma(3, 0.2387)$, leading to $Var(G) = 52.7$, which is now in good agreement with simulations. It thus appears that the "backward" observation shortening also affects the observed serial intervals and the difference between observed generation time and serial interval distributions. In this case, in order to theoretically predict the observed difference in variances, one would have to calculate the effect of "backward" observation of a generation time on the marginal distribution of the corresponding latent and incubation periods.

Finally, it may be interesting to investigate how large the quotient between the coefficient of variation of S and that of G (the parameter c in a previous Section) is as a consequence of model assumptions. Since the means of G and S are the same, we will have $c^2 =$

$Var(S)/Var(G)$ and thus $c^2 = 78.99/75 = 1.053$ according to the above calculations. Thus $c = 1.026$ in the simulation model.

7 Estimating the growth rate

Estimating the growth rate of the outbreak or its doubling time or other equivalent measures (under the assumption of exponential growth) is interesting per se and is useful for predictions of the future size of the outbreak if it is assumed that the current growth rate will not change. There are several possible methods but, unfortunately, it seems difficult to evaluate them theoretically on finite samples. We now concentrate on estimating the growth rate directly from case data. However, there seems to be a lack of systematic evaluations adapted to infectious disease spread data. A sensible approach is then to simulate the performance of the chosen estimation method in simulations as close as possible to the data generating situation. We have therefore evaluated various data-based methods in our simulation model. The compared methods are:

a) linear regression on logarithms of cumulative numbers of notified cases,

b) linear regression on logarithms of daily numbers of notified cases,

c) taking the mean of daily ratios of successive cumulative numbers,

d) estimating $exp(r)$, the daily multiplication factor, with a branching process type estimator of the form $(n(2) + \ldots . + n(K))/(n(1) + \cdots + n(K-1))$, where $n(i)$ is the number of cases notified day i, and reporting the logarithm,

e) fitting the discretized renewal equation described in Section 2 of the main paper to observed incidence data, using the generation time distribution estimated from backward times. This method produces an estimate of R_0 and not of the growth rate r but can anyway extrapolate values of future incidence.

Using the 1000 simulated epidemic trajectories, the estimators a) - e) of the exponential growth rate r and their usefulness in predicting the epidemic size 6 weeks after reaching 4500 notified cases were tested. The methods a) - d) estimate r using data from the last 6 weeks before reaching level 4500, while the fifth method, based on the discretized renewal equation (Eq. (5), Section 2 of main paper) estimates R_0, using regression weights derived from the estimated generation time distribution based on observed backward serial intervals, as suggested in [1].

In the simulation model, the true value of r is 0.03870 and of $exp(r)$ is 1.03946. It should be noted that what is then used in the prediction of the situation 6 weeks later would be the factor $exp(42r)$ (with true value = 5.07973), which is also studied, both in isolation and used as predictor in combination with the last datum.

All 4 estimators of r show reasonably concentrated values around the true value 0.0387, as shown in Table 2:

However, all slightly overestimate r, since the 95% confidence intervals do not contain the true value, although by less than 1%, on average. If one considers the prediction factor $exp(42r)$, on average all four estimators again overestimate a little. Estimator (a)

Table 2: Some distributional summaries for the estimators a)-d) (see text) of the exponential increase rate based on time series of notified cases. The theoretical value to be estimated is $r = 0.03870$.

Statistic	(a)	(b)	(c)	(d)
Maximum	0.04588	0.04605	0.04583	0.05168
Median	0.03883	0.03901	0.03885	0.03904
Minimum	0.03272	0.03125	0.03319	0.02785
**Mean**	**0.03891**	**0.03905**	**0.03892**	**0.03901**
Std Dev	0.00220	0.00230	0.00217	0.00407
Upper 95% Mean	0.03905	0.03919	0.03905	0.03927
Lower 95% Mean	0.03877	0.03890	0.03878	0.03876

is the best, with mean 5.14729, but the *sd* of the distribution is now 0.48000 and a 95% prediction interval ranges from 4.2914 to 6.1641, which means $[-16\%, +21\%]$ relative to the true value.

However, if one implements the prediction by multiplying the last cumulative datum by the estimate of $exp(42r)$ and divides the result by the true cumulative datum 42 days later, there is still overestimation, by about 1%, but the prediction interval for method (a) has shrunk a little, to $[-13\%, +18\%]$. There is no big difference between methods, but (a) seems to have a slight advantage.

Finally, we study the performance of the renewal equation approach prposed in [1]. This approach is intended to allow estimation of R_t (in our simulation, R_t is kept constant $= R_0$ all the time and the method is adapted accordingly) and to further allow prediction of cases 6 weeks after the last datum. There are many small details to decide when using this method. We have made the following assumptions and considerations:
- the time series of reported cases starts with day 1 when the first case is reported (= becomes symptomatic in our simulation) and goes on until the day the total 4500 is reached. The length of this series is thus different from the one counted from the introduction of the first infective.
- the method uses the serial interval distribution, assumed Gamma, as estimated from data. However, this distribution has to be discretized to daily probabilities. We know from previous discussions that this distribution is a biased estimate of the true generation time distribution.
- assuming the auto-regressive Poisson model for the time series of daily cases, the estimator of R_0 can be explicitly deduced.
- with this estimated R_0, the time series can be brought forward until the desired prediction date is reached.

The results indicate that this method underestimates R_0 but has good predictive power anyway, as follows:
-while the true value of R_0 in the simulation is 1.7, the mean of estimates obtained is 1.566 with 95% confidence limits 1.564 and 1.568, the minimum value in 1000 simulations was 1.467 and the maximum 1.683;
- using the quotient predicted/true observed value 6 weeks later as indicator of predictive accuracy, the mean was 1.0040 with 95% confidence limits 1.0006 and 1.0074, with minimum value 0.856 and maximum 1.213.

Thus, the predictor is almost unbiased and a 95% prediction interval is [-10%,+12%] around the true value, which is slightly better than the previous r-based methods.

8 References

1. WHO Ebola Response Team (2014). Ebola virus disease in West Africa – the first 9 months of the epidemic and forward projections. N Engl J Med. 371: 1481-1495.

2. Jagers P. (1975) Branching processes with biological applications. John Wiley & Sons.

3. Svensson, Å. (2007) A note on generation times in epidemic models. Math. Biosci. 208: 300-311.

4. Phillips D.T. and Beightler C. (1971), Procedures for generating gamma variates with non-integer parameter sets. WSC '71 Proceedings of the 5th conference on Winter simulation, p 421-427, ACM New York, NY, USA.

5. Scalia Tomba, G., Svensson, Å., Asikainen, T. and Giesecke, J. (2010). Some model based considerations on observing generation times for communicable diseases. Math. Biosci. 223: 24-31.

6. Champredon, D. and Dushoff, J. (2016). Intrinsic and realized generation intervals in infectious-disease transmission. Proc. R. Soc. B, 282: 2015-2026.
